# Comparison of Length of Treatment and Comorbidities in Older and Adult Hospitalized Patients with Bacterial Community-Acquired Pneumonia

**DOI:** 10.5152/eurasianjmed.2025.25910

**Published:** 2025-10-30

**Authors:** Muhammad Fachri, Mochammad Hatta, Dinda Zakia, Risky Akaputra, Atthariq Wahab, Azhar Azhar, Ade Rifka Junita

**Affiliations:** 1 Department of Pulmonology and Respiratory Medicine, Muhammadiyah University Faculty of Medicine and Health, Jakarta, Indonesia; 2 Department of Molecular Biology and Immunology, Hasanuddin University Faculty of Medicine, Makassar, Indonesia; 3 Department of Clinical Microbiology, Hasanuddin University Faculty of Medicine, Makassar, Indonesia; 4 Medical Study Program, Muhammadiyah University Faculty of Medicine and Health, Jakarta, Indonesia; 5 Department of Community Medicine, Muhammadiyah University Faculty of Medicine and Health, Jakarta, Indonesia; 6 Department of Pulmonology and Respiratory Medicine, Hasanuddin University Faculty of Medicine, Makassar, Indonesia

**Keywords:** Adults, bacterial community-acquired pneumonia, comorbidities, length of treatment, older

## Abstract

**Background:**

Among the 1 017 290 people surveyed, the prevalence of pneumonia was 2.5% in the 55-64 age group, 3.0% in the 65-74 age group, and 2.9% in those aged 75 years and older. This indicates an increase in pneumonia prevalence from 1.8% to 2% in Indonesia. This study aimed to compare the length of treatment and comorbidities in older and adult patients with community-acquired pneumonia at Jakarta Sukapura Islamic Hospital and Jakarta Pondok Kopi Islamic Hospital between September 2022 and September 2024.

**Methods:**

This was a comparative study with a cross-sectional design, and secondary data from 244 patient samples were used.

**Results:**

Among adult patients, 101 had a length of treatment of ≤5 days, whereas 17 had a length of treatment of >5 days. Among older patients, 67 had a length of treatment of ≤5 days, whereas 59 had a length of treatment of >5 days. Comorbidities were absent in 69 adult and 45 older patients. A total of 49 adult patients and 81 older patients had comorbidities.

**Conclusion:**

Older patients with community-acquired pneumonia had a longer treatment duration and more comorbidities than adult patients with bacterial community-acquired pneumonia.

**Main Points**

- Male patients have a higher incidence of community-acquired pneumonia than females.
- Older patients have a longer treatment duration than adult patients.
- Comorbidities are more common in older adults than in adult patients.
- Patients with comorbidities require longer treatment than those without.
- Occupational factors and working conditions influence pneumonia risks.

## Introduction

Community-acquired pneumonia is an acute inflammatory condition that affects the lung parenchyma, caused by infection with pathogenic microorganisms acquired outside healthcare facilities. The main risk factors include extreme age (both older adults and infants) and unhealthy lifestyle habits (e.g., smoking and alcohol consumption). Pneumonia is among the most significant global health challenges, especially considering its high prevalence and impact on the older population. The later phase of life is characterized by a decline in physiological capacity, reducing the body’s ability to maintain homeostasis when exposed to various stressors. According to the Ministry of Health, adulthood begins at 19-59 years of age.^3^

Lower respiratory tract infections, particularly pneumonia, often occur in older individuals and have a high mortality rate. The causes include a decline in immunological processes. With increasing age, lung function decreases, marked by reduced respiratory system elasticity. This is caused by increased chest wall stiffness, which facilitates the inhalation and retention of microorganisms in the respiratory tract, whereas ciliary clearance decreases.^4^

Therefore, a strong correlation is observed between aging and decline in general organ function, physical function, barrier integrity, pathogen defense, and immune system efficiency. These factors make older patients more susceptible to community-acquired pneumonia and pneumonia caused by multidrug-resistant bacteria.^5^

Before the introduction of antibiotics, *Streptococcus pneumoniae* (pneumococcus) was responsible for 90%-95% of infections. Increased antibiotic use and pneumococcal vaccination have reduced this prevalence to 5%-15% in recent United States studies and 20%-25% in European studies.^6^ A meta-analysis pooling data from 31 studies to examine the role of viruses in community-acquired pneumonia identified rhinoviruses and influenza as the most common causes. Other viruses, such as coronavirus, parainfluenza, adenovirus, and human metapneumovirus, were less common but still posed a significant risk, affecting 1%-4% of patients. In almost all studies, these etiologies were difficult to identify, although bacterial causes were more common.^7^

Pneumonia was among the 10 most common causes of hospitalization in Indonesia in 2020. Globally, it ranks as the eighth leading cause of death in the United States and imposes a significant economic burden on healthcare systems. Long-term antibiotic use also results in high treatment costs, even in patients without significant risks or comorbidities. Therefore, the rationale for antibiotic use in pneumonia treatment warrants further consideration.^8^

Data from the 2018 Indonesian Basic Health Research (Riskesdas) showed a pneumonia prevalence of 2.21% across all age groups, with an increasing trend among older individuals: 2.5% in the 55-64 age group, 3.0% in the 65-74 age group, and a slight decrease to 2.9% in those aged 75 years and older. Among the 1 017 290 individuals surveyed, pneumonia cases reached 2.5% in the 55-64 age group, 3.0% in the 65-74 age group, and 2.9% in those aged 75 years and older. These findings indicate an increase in pneumonia prevalence in Indonesia from 1.8% to 2%, according to the 2018 Riskesdas data.^9^ A study analyzing data from 3 Southeast Asian countries (Malaysia, Indonesia, and the Philippines) found that community-acquired pneumonia was one of the most common causes of hospitalization, accounting for 1.5%-19.9% of all hospitalizations, with mortality rates ranging from 1.4% to 4.2%.^10^ Based on these conditions, a study that discusses the comparison of the duration of treatment and the condition of comorbidities in elderly bacterial community pneumonia patients with adult bacterial community pneumonia patients in hospital inpatients was to be conducted.

## Materials and Methods

### Study Design

This analytical observational study employed a comparative approach to assess the length of treatment and comorbidities in patients with bacterial community-acquired pneumonia. Patients were classified into 2 groups: older and adult groups, who were treated in the inpatient wards of Jakarta Sukapura Islamic Hospital and Jakarta Pondok Kopi Islamic Hospital. The study used a cross-sectional design and relied on secondary data from patient medical records collected between September 2022 and September 2024. The study population comprised older and adult patients with bacterial community-acquired pneumonia admitted to Jakarta Sukapura Islamic Hospital and Jakarta Pondok Kopi Islamic Hospital during this period. A total sampling method was used, in which all eligible patients in the population were included as study participants.

### Statistical Analysis

Univariate analysis was conducted to describe the percentage distribution of each characteristic variable among older and adult patients with bacterial community-acquired pneumonia, including age, sex, and occupation. Bivariate analysis was performed using the chi-square and Mann–Whitney tests. The chi-square test was used to assess the relationship between the length of treatment and comorbidities in older and adult patients with bacterial community-acquired pneumonia, whereas the Mann–Whitney test was used to determine whether there were statistically significant differences between the 2 groups regarding the length of treatment and comorbidities.

### Ethics

This study was approved by the Ethics Committee of the Muhammadiyah University Faculty of Medicine and Health (Approval no.173/PE/KE/FKK-UMJ/X1/2024; Date: November 01, 2024). All participants or their parents/guardians gave their informed consent, and the study was approved by the review boards of the participating institutes.

## Results

Table 1 presents the frequency and percentage distribution of age characteristics among adult and older patients with bacterial community-acquired pneumonia. The data indicate that older patients (>60 years) comprised 126 cases (51.6%), whereas adult patients (19-59 years) accounted for 118 cases (48.4%).

For the frequency and percentage of gender in adult bacterial community-acquired pneumonia patients with elderly and adult bacterial community-acquired pneumonia patients, the most common gender is male, with 66 adult patients (47.5%) and 73 older patients (52.5%), while the most common gender is female, with 52 adult patients (49.5%) and 53 older patients (50.5%).

For occupational distribution, the most common category among adult patients with bacterial community-acquired pneumonia was “occupation unknown or not having a permanent job” (47 patients, 42.7%), followed by housewives (26 patients, 36.0%), private employees (20 patients, 90.9%), self-employed individuals (12 patients, 73.3%), freelancers (4 patients, 44.4%), students (4 patients, 100.0%), civil servants (3 patients, 75.0%), retirees (1 patient, 20.0%), and unemployed individuals (1 patient, 25.0%). Among older patients, the most common category was also “occupation unknown” (63 patients, 57.3%), followed by housewives (38 patients, 64.0%), freelancers (5 patients, 55.6%), self-employed individuals (6 patients, 26.7%), retirees (6 patients, 80.0%), unemployed individuals (5 patients, 75.0%), private employees (2 patients, 9.1%), and civil servants (1 patient, 25.0%).

The most common comorbidity among both adult and older patients was hypertension, affecting 31 adult and 45 older patients. The second most common comorbidity was diabetes mellitus, reported in 22 adult and 30 older patients.

In the chi-square test results presented in Table 2, among patients without comorbidities, 91 (79.8%) had a length of treatment of ≤5 days, whereas 23 (20.2%) had a length of treatment of >5 days. Among patients with comorbidities, 77 (59.2%) had a length of treatment of ≤5 days, whereas 53 (40.8%) had a length of treatment of >5 days. These findings indicate that patients with comorbidities required longer treatment duration than those without comorbidities.

Table 3 shows that among adult patients, 101 (60.1%) had a length of treatment of ≤5 days, whereas 17 (22.4%) had a length of treatment of >5 days. Among older patients, 67 (39.9%) had a length of treatment of ≤5 days, whereas 59 (77.6%) had a length of treatment of >5 days.

The results in Table 4 also indicate a significant difference in treatment duration between adult and older patients. The mean rank for adult patients was 102.08, whereas that for older patients was 141.63, indicating that older patients generally required longer treatment durations than adult patients.

Table 5 shows that among patients without comorbidities, 69 (60.5%) were adults, whereas 45 (39.5%) were older individuals. In contrast, among patients with comorbidities, 49 (37.7%) were adults, whereas 81 (62.3%) were older individuals. These findings indicate a relationship between comorbidities and age.

Table 6 presents treatment duration stratified by comorbidity status. Among adult patients without comorbidities, 62 (89.9%) had a length of treatment of ≤5 days, whereas 7 (10.1%) had a length of treatment of >5 days. Among adult patients with comorbidities, 39 (79.6%) had a length of treatment of ≤5 days, whereas 10 (20.4%) required >5 days. Among older patients without comorbidities, 29 (64.6%) had a length of treatment of ≤5 days, whereas 16 (35.6%) required >5 days. However, among older patients with comorbidities, 38 (46.9%) had a length of treatment of ≤5 days, whereas 43 (53.1%) had a length of treatment of >5 days.

## Discussion

The results of this study indicate that community-acquired pneumonia was more prevalent in older patients, with 126 cases (51.6%) compared to 118 cases (48.4%) of adult patients. These findings are consistent with those reported by Ramirez et al^11^ (2017), who reported an incidence of 2093 cases per 100 000 people in older patients, compared to approximately 706 cases per 100 000 people in adult patients. This difference may be related to age-related declines in pulmonary physiology, which make it more difficult for older patients to combat bacterial lung infections.^4^

The increased risk of community-acquired pneumonia in the older population is associated with several factors. For example, nasal mucociliary clearance is less effective in older than in younger people. Studies on both animals and humans have demonstrated that aging impairs pulmonary defense mechanisms, such as mechanical resistance, phagocytic activity, and humoral immunity, as well as reductions in T and B cell functions. Additionally, oropharyngeal bacterial colonization is more common in older patients.^12^

Besides age, community-acquired pneumonia can be influenced by other factors, including sex. Higher smoking rates among men have been associated with a higher incidence of community-acquired pneumonia compared to women.^13^ In this study, the proportion of male patients was higher in both the adult (60; 47.6%) and older (66; 52.4%) groups, whereas female patients accounted for 52 (49.5%) of the adult cases and 53 (50.5%) of the older cases. These findings are consistent with those of Andayani^14^ (2014), who reported a higher incidence in men (65.0%) than that in women (35.0%).

Regarding occupational characteristics, the most common occupation among adult patients was housewives (26 patients, 36%), followed by private employees (20 patients, 90.9%). Among older patients, housewives were the most common occupation (38 patients, 64.0%), followed by retirees (6 patients, 80.0%). Almirall et al^15^ (2015) found that office work was considered a protective factor against community-acquired pneumonia, whereas construction work posed a higher risk. However, this association was no longer significant after adjusting for working conditions. Although certain occupations may not directly increase the risk of community-acquired pneumonia, certain working conditions, such as exposure to dust and sudden temperature changes, are preventable risk factors. Therefore, workplace preventive measures, such as the use of personal protective equipment and regulations that ensure environmental stability, may help reduce the risk of community-acquired pneumonia.^15^

The most common comorbidities among adult and older patients with bacterial community-acquired pneumonia in this study were hypertension, affecting 31 (40.8%) and 45 (59.2%) patients, respectively, followed by diabetes mellitus, affecting 22 (42.3%) adult and 30 (57.7%) older patients. Similarly, Rivero-Calle et al^16^ (2016) identified metabolic disease (27.4%), cardiovascular disease (17.8%), and diabetes (15.5%) as major risk factors for pneumonia in adult patients, particularly those over 55 years of age.

Older individuals, especially those aged >65 years, are more likely to develop chronic diseases because of their limited regenerative capacity and increased susceptibility to diseases, syndromes, and injuries than younger adults. Age-related structural, functional, and molecular physiological changes affect various systems, with nervous system changes often leading to cognitive impairment and cardiovascular changes resulting in high blood pressure and lower cardiac output. Additionally, respiratory system functions decline, leading to decreased arterial oxyhemoglobin levels. Alsuwaidan et al^17^ reported that comorbidities in older patients (>65 years) in Saudi Arabia are associated with various chronic diseases that require close monitoring during treatment.

In the above study, patients without comorbidities but with a length of treatment of ≤5 days accounted for 84 (79.2%) cases, whereas those with a length of treatment of >5 days were 22 (20.8%) cases. Among patients with comorbidities, 76 (60.8%) had a length of treatment of ≤5 days, whereas 49 (39.2%) required >5 days. In addition, in adult patients, the frequency of length of treatment ≤5 days was 96 (60.0%), whereas 16 (22.5%) required >5 days. In older patients, 64 (40.0%) had a length of treatment of ≤5 days, whereas 55 (77.5%) required >5 days. These findings indicate that patients with comorbidities required longer treatment than those without comorbidities, and older patients with community-acquired pneumonia required a longer treatment duration than adult patients. Comorbidities also influence the etiology of community-acquired pneumonia. Gutiérrez et al^18^ (2005) found that bacterial infections occurred more frequently in patients with comorbidities (65, 58.6%) than in those without (45, 40.5%). This suggests that bacterial community-acquired pneumonia is more common in patients with comorbidities.^18^

These findings align with those by Suter-Widmer et al^19^ (2012), who reported an average length of treatment of 9.8 days in a population with a mean age of 72 years and a high burden of comorbidities. In addition, younger patients exhibited a more pronounced inflammatory response yet required a shorter length of treatment than older patients. This suggests that older patients with comorbidities tend to require a longer treatment duration than adult patients.^19^

A limitation of this study is that the medical records at Jakarta Pondok Kopi Islamic Hospital are not yet in electronic form. Consequently, data collection was time-consuming, and some records were incomplete, missing, or not sequential. In addition, the study duration was relatively short, and obtaining permission to conduct research at Jakarta Sukapura Islamic Hospital took nearly 2 weeks, reducing the time available for data collection and leading to some missing data. This study revealed a significant association between patient age and length of treatment, with older patients requiring longer treatment durations than adult patients. Additionally, a significant relationship was observed between comorbidities and age, as older patients had a higher prevalence of comorbidities than adult patients.

The results of this study can help in clinical practice in managing community-acquired pneumonia patients, specifically in the management of community-acquired pneumonia patients, which requires a longer time than managing adult community-acquired pneumonia patients. This is because the immunity of elderly patients has decreased. Additionally, the management of elderly community-acquired pneumonia patients with comorbidities takes longer than the management of adult community-acquired pneumonia patients. This indicates that, in clinical practice, the management of elderly community-acquired pneumonia patients with comorbidities has a worse prognosis compared to the management of managing adult community-acquired pneumonia patients with comorbidities. In administering antibiotics for the management of community-acquired pneumonia patients, especially those who are of older age and have comorbidities, a policy must be implemented based on the results of microorganism cultures and antibiotic sensitivity tests, even potentially using higher antibiotic levels. This will impact on the duration of treatment positively. The policy for managing community-acquired pneumonia patients based on the results of microorganism cultures and antibiotic sensitivity tests must apply to all types of community-acquired pneumonia patients, whether elderly or adults, with or without comorbidities.

The results of this study found a significant comparison between the length of treatment and the age of the patient, where in elderly patients the length of treatment was longer than in adult patients. Additionally, it was also found that there was a significant comparison between comorbidities and age, where elderly patients had more comorbidities than adult patients.

## Figures and Tables

**Table 1.** Distribution of Characteristics of Adult and Older Patients with Bacterial Community-Acquired Pneumonia

Characteristics		Adult Patients		Older Patients	
		Frequency	%	Frequency	%
Age	19-60 years >60 years	118 0	48.4 0	0 126	0 51.6
Gender	Male Female	66 52	47.5 49.5	73 53	52.5 50.5
Occupation	Unemployed Housewife Self-employed Private employee Freelancer Government employee Retired Student Occupation unknown	1	25.0	5	75.0
		26	36.0	38	64.0
		12	73.3	6	26.7
		20	90.9	2	9.1
		4	44.4	5	55.6
		3	75.0	1	25.0
		1	20.0	6	80.0
		4	100.0	0	0.0
		47	42.7	63	57.3
Comorbidities	Asthma Heart disease Hypertension Diabetes mellitus Pulmonary TB Extrapulmonary TB Stroke	8	42.1	11	57.9
		7	33.3	14	66.7
		31	40.8	45	59.2
		22	42.3	30	57.7
		5	55.6	4	44.4
		0	0.0	1	100.0
		0	0.0	3	100.0

TB, tuberculosis.

**Table 2.** Relationship Between Length of Treatment and Comorbidities in Adult and Older Patients with Bacterial Community-Acquired Pneumonia

Chi-Square Test	Length of Treatment				Total		OR (95% CI)	*P*
	**≤5 days**		**>5 days**					
	**n**	**%**	**N**	**%**	**n**	**%**		
Without comorbidities	91	79.8	23	20.2	114	100.0	2.723	<.001
With comorbidities	77	59.2	53	40.8	130	100.0	1.5-4.8	
**Total**	**168**	**68.9**	**76**	**61.0**	**244**	**100.0**		

CI, confidence interval; OR, odds ratio.

**Table 3.** Differences in Length of Treatment in Adult and Older Patients with Bacterial Community-Acquired Pneumonia

Chi-Square Test	Age Group				Total		OR (95% CI)	*P*
	**Adult Patients**		**Older Patients**					
	**n**	**%**	**n**	**%**	**n**	**%**		
Length of treatment ≤ 5 days	101	60.1	67	39.9	168	100.0	5.232	<.001
Length of treatment > 5 days	17	22.4	59	77.6	76	100.0	2.8-9.7	
**Total**	**118**	**48.4 **	**119**	**30.7 **	**244**	**100.0 **		

CI, confidence interval; OR, odds ratio.

**Table 4.** Comparison of Length of Treatment Between Adult and Older Patients with Bacterial Community-acquired Pneumonia

Age Group	Frequency	Mean Rank	Mann–Whitney *U*	Sum of Ranks	*Z*	*P*
Adult patients (19-59)	118	102.08	5 024.000	12 045.00	−5.453	<.001
Older patients (≥60)	126	141.63		17 845.00		
**Total**	**244**					

**Table 5.** Relationship Between Comorbidities in Adult and Older Patients with Bacterial Community-Acquired Pneumonia

Chi-Square Test	Age Group				Total		OR (95% CI)	*P*
	Adult Patients		Older Patients					
	**n**	%	n	%	n	%		
Without comorbidities	69	60.5	45	39.5	114	100.0	2,535	<.001
With comorbidities	49	37.7	81	62.3	130	100.0	1.5-4.2	
**Total**	**112**	**48.5 **	**119**	**51.5 **	**244**	**100.0 **		

CI, confidence interval; OR, odds ratio.

**Table 6.** Relationship Between Comorbidities and Length of Treatment in Older and Adult Patients with Bacterial Community-Acquired Pneumonia

	Chi-Square Test	Length of Treatment				Total		OR (95% CI)	*P*
		≤5 days		>5 days					
		n	%	n	%	n	%		
Adult patients	Without comorbidities	62	89.9	7	10.1	69	100.0	2.271	.118
	With comorbidities	39	79.6	10	20.4	49	100.0	0.7– 6.4	
Older patients	Without comorbidities	29	64.6	16	35.6	45	100.0	2.051	0.05
	With comorbidities	38	46.9	43	53.1	81	100.0	0.9-4.3	
	**Total**	**168**	**68.9**	**76**	**31.1 **	**244**	**100.0 **		

CI, confidence interval; OR, odds ratio.

## Data Availability

The data that support the findings of this study are available on request from the corresponding author.
